# Design of a Synthetic Long Peptide Vaccine Targeting HPV-16 and -18 Using Immunoinformatic Methods

**DOI:** 10.3390/pharmaceutics15071798

**Published:** 2023-06-23

**Authors:** Alexandru Tîrziu, Speranța Avram, Leonard Madă, Mihaela Crișan-Vida, Casiana Popovici, Dan Popovici, Cosmin Faur, Corina Duda-Seiman, Virgil Păunescu, Corina Vernic

**Affiliations:** 1 Department of Functional Sciences, “Victor Babes” University of Medicine and Pharmacy, Eftimie Murgu Square, No. 2, 300041 Timisoara, Romania; alexandru.tirziu@umft.ro (A.T.); cvernic@umft.ro (C.V.); 2 Department of Anatomy, Animal Physiology and Biophysics, Faculty of Biology, University of Bucharest, 91-95 Splaiul Independentei, 050095 Bucharest, Romania; speranta.avram@gmail.com; 3 Syonic SRL, Grigore T Popa Street, No. 81, 300254 Timisoara, Romania; leo.mada@syonic.eu; 4 Department of Automation and Computers, Politehnica University of Timisoara, 300006 Timisoara, Romania; mihaela.vida@upt.ro; 5 Section of Bioinformatics, Division of Systems Medicine, Department of Metabolism, Digestion and Reproduction, Imperial College London, South Kensington Campus, London SW7 2AZ, UK; c.popovici20@imperial.ac.uk; 6 Department of Mathematics, University of the West Timişoara, Bd. Vasile Pârvan No. 4, 300223 Timişoara, Romania; dan.popovici@e-uvt.ro; 7 Department of Orthopaedic Surgery, University of Medicine and Pharmacy “Victor Babes”, Dropiei Street, No. 7, sc B, ap 8, 300661 Timisoara, Romania; faur.cosmin@umft.ro; 8 Department of Chemistry and Biology, Faculty of Chemistry, Biology, Geography, West University of Timisoara, 16 Pestalozzi, 300115 Timisoara, Romania; corina.duda@e-uvt.ro; 9 Center for Gene and Cellular Therapies in the Treatment of Cancer Timisoara-OncoGen, Clinical Emergency County Hospital “Pius Brinzeu” Timisoara, No. 156 Liviu Rebreanu, 300723 Timisoara, Romania; 10 Immuno-Physiology and Biotechnologies Center, Department of Functional Sciences, “Victor Babes” University of Medicine and Pharmacy, No. 2 Eftimie Murgu Square, 300041 Timisoara, Romania; 11 Discipline of Medical Informatics and Biostatistics, “Victor Babes” University of Medicine and Pharmacy, 300041 Timisoara, Romania

**Keywords:** human papillomavirus, cervical cancer, therapeutic vaccine, epitopes, molecular docking, in silico, synthetic long peptides

## Abstract

Human papillomavirus types 16 and 18 cause the majority of cervical cancers worldwide. Despite the availability of three prophylactic vaccines based on virus-like particles (VLP) of the major capsid protein (L1), these vaccines are unable to clear an existing infection. Such infected persons experience an increased risk of neoplastic transformation. To overcome this problem, this study proposes an alternative synthetic long peptide (SLP)-based vaccine for persons already infected, including those with precancerous lesions. This new vaccine was designed to stimulate both CD8+ and CD4+ T cells, providing a robust and long-lasting immune response. The SLP construct includes both HLA class I- and class II-restricted epitopes, identified from IEDB or predicted using NetMHCPan and NetMHCIIPan. None of the SLPs were allergenic nor toxic, based on in silico studies. Population coverage studies provided 98.18% coverage for class I epitopes and 99.81% coverage for class II peptides in the IEDB world population’s allele set. Three-dimensional structure ab initio prediction using Rosetta provided good quality models, which were assessed using PROCHECK and QMEAN4. Molecular docking with toll-like receptor 2 identified potential intrinsic TLR2 agonist activity, while molecular dynamics studies of SLPs in water suggested good stability, with favorable thermodynamic properties.

## 1. Introduction

Cervical cancer represents the fourth most common neoplastic lesion among women worldwide [1], with an estimated 604,000 new cases and 342,000 deaths in 2020. 90% of newly diagnosed cancers, with most deaths occurring in middle- and low-income countries [2]. The human papillomavirus (HPV) infection is responsible for most cases [3]. Besides cervical carcinoma, HPV is also responsible for uterine pre-neoplastic lesions (cervical intraepithelial neoplasia, CINs) that require a rigorous screening process through cytological examination and prompt therapeutic interventions [4].

Human papillomaviruses (HPVs) are double-stranded, circular DNA viruses with high epithelial tropism. The 48 species and 206 genotypes are clinically classified based on their carcinogenic potential: high risk (16, 18, 31, 33, 35, 39, 45, 51, 52, 56, 58, 59, 68, 73, 82) and low risk [5]. While high-risk types present a more aggressive behavior that leads to neoplastic transformation, low-risk HPVs give rise to benign papillomatous lesions, such as anogenital warts (condyloma acuminata). Among the high-risk species, HPV-16 and HPV-18 are the most prevalent ones, being responsible for up to 77% of all cervical cancers [1,5,6]. The prevalence of the other genotypes is far lower: in Europe, HPV-33 and HPV-45 are ranked the 3rd and 4th most prevalent, with a prevalence in carcinomas of only 4.4% and 4.3%, respectively [6].

The HPV genome is a circular, double-stranded DNA that encodes 6 early genes (E1, E2, E4, E5, E6, and E7) and 2 late genes (L1 and L2) [7]. A non-coding region that contains the early promoter and regulatory elements plays a major role in viral replication [8].

E6 and E7 proteins promote tumorigenesis by disrupting the apoptotic pathways while promoting angiogenesis, invasion, metastasis, and telomerase activity.

E6 interacts with E6AP to form a complex that degrades p53, allowing cells to bypass cell cycle checkpoints and proliferate uncontrollably. Moreover, p53 degradation also leads to underexpression of thrombospondin-1 and maspin, and overexpression of HIF-1α and IL-8, promoting blood vessel formation. 

E7 degrades pRb, activating E2F and promoting premature entry into the S phase of the cell cycle. Enhanced telomerase activity occurs through the upregulation of hTERT and degradation of NFX1 (hTERT repressor), leading to increased telomere replication. E6 and E7 facilitate invasion and metastasis by activating transcription factors involved in epithelial-mesenchymal transition, such as Slug, Twist, and ZEB1/2 [9].

E6- and E7-dependent downregulation of MHC class I and class II molecules, E-cadherin, and CCL20 [10] leads to (1) a reduction in epitope presentation by dendritic cells and antigen recognition by T cells; (2) impairment of antigen-presenting cell (APC)-infected keratinocyte interaction; and (3) loss of positive chemotactic signals for Langerhans cells (LC). Consequently, viral DNA integration into the host genome, with subsequent nucleic acid replication and transcription, is achieved in the presence of a hampered immune response [11].

Compared to healthy keratinocytes, (pre)malignantly transformed cells express E6 and E7 early proteins, making them suitable candidates for protein/peptide-based therapeutic vaccines [10].

Prevention is key for HPV-related illnesses and involves proper sexual hygiene, along with vaccination. The current vaccination platform utilizes virus-like particles (VLPs) containing the highly immunogenic L1 capsid protein to produce high titers of protective neutralizing antibodies against future HPV infections. The three commercially available vaccines, Gardasil (against HPV-16 and -18), Cervarix (against HPV 6-, 11-, 16-, and -18), and Gardasil-9 (HPV-6, 11, 16, 18, 31, 33, 45, 52, 58) [12] provide almost 100% protection against high-risk viral types in women between the ages of 9 and 26 years old [5]. However, the vaccine is ineffective against a preexisting infection, which greatly limits its usefulness in adults who have already started their sexual life. The poor vaccine availability in developing countries, exacerbated by the COVID-19 pandemic, can be attributed to low cost-effectiveness, a worldwide shortage of HPV vaccine doses, and a deficit of medical and administrative personnel [13]. In addition, low vaccination rates among boys and men contribute to the unsuccessful decline of skin and mucosal cancers worldwide [14].

The three available vaccines exert a highly potent prophylactic effect by interfering with the adhesion of HPV to keratinocytes, thus preventing the infection and disabling the pathway to malignant keratinocyte transformation (primary prevention). However, vaccination has no effect on persistent infection due to the lack of L1 antigen expression in basal and neoplastic epithelial cells, and, as a consequence, subsequent DNA integration may lead to malignant transformation [15].

Once malignant transformation occurs, cervical cancer treatment strategies vary from surgical resection to radio chemotherapy and immunotherapy, based on the level of dysplasia, tumor dimensions, lymph node invasion, and distant site metastasis. All therapeutic actions present a high risk of adverse effects (including psychosocial and psychosexual problems) and do not guarantee total tumor cell clearance [8]. Furthermore, as chemotherapy is non-specific, it often causes treatment-associated cytotoxicity [16].

Therapeutic HPV vaccination can be employed to cure the infection (secondary prophylaxis) or to target malignant tumors. This approach stimulates the differentiation of naïve T cells into effector CD8+ cytotoxic T cells and CD4+ T helper cells. The result is a CD8+-dependent cytotoxicity against virally infected or malignantly transformed keratinocytes, with increased pro-inflammatory cytokine release by the CD4+ Th1 cells. The objective during secondary prophylaxis is the full resolution of the HPV infection before any malignant transformation occurs.

By using minimal antigenic components, peptide-based vaccination allows a safer and more controlled targeted immune response.

Peptide-based vaccines express increased stability during storage and transport, while being easily synthesized with high purity and yield via chemical or biological methods [17]. Although their immunogenicity and stability may be poor in vivo, these caveats can be overcome by combining multiple peptides into a single construct, and by simultaneous administration of adjuvants such as toll-like receptor agonists.

Peptide-based strategies utilize either highly conserved epitopes (overcoming the effect of point mutations on antigen presentation) or a variety of highly immunogenic antigenic determinants. Single epitope strategies are mainly based on CD8+-restricted epitopes that can be easily cleaved by extracellular peptidases. To improve their stability and potentiate the immune response, CD8+-restricted epitopes can be combined with CD4+-restricted epitopes in synthetic long peptide constructs. The result is a potent, specific, cytotoxic T cell response against virally infected cells, amplified by the CD4+-mediated pro-inflammatory cytokine secretion.

The combination of an HLA class I-restricted epitope with an HLA class II-restricted epitope and a cleavable linker provides a bidirectional stimulation of the T cell population via canonical antigen presentation (HLA class II-CD4+ cell interaction), along with the cross-presentation of class I-restricted epitopes to CD8+ T cells. 

In this study, we present the design of a therapeutic synthetic long peptide-based vaccination platform, targeting patients with persistent HPV-16 and HPV-18 infections using an immunoinformatic approach.

## 2. Materials and Methods

### 2.1. HPV-16 and -18 Peptide Identification from the IEDB Database

Epitopes from E6 and E7 proteins belonging to HPV-16 and -18 types were extracted from the publicly available Immune Epitope DataBase (IEDB), which contains approximately 260,000 antigenic sequences collected from over 20,000 curated, peer-reviewed publications [18]. The search criteria included: (a) linear peptides; (b) belonging to HPV-16 (*Alphapapillomavirus* 9) or HPV-18 (*Alphapapillomavirus* 7); (c) the host was selected to be human; (d) the disease was selected to be infectious; (e) T cell-based assays.

### 2.2. Neo-Antigen Prediction Using Artificial Neural Networks

To enhance the recognition repertoire, neo-epitopes were predicted using an artificial neural network-based model from IEDB Tools.

IEDB Tools provides 2 consensus ANN-based algorithms that predict epitopes present in a specific protein with a high probability to bind to a user-specified HLA allele dataset. Class I and class II prediction tools estimate, for each peptide, an IC50 and a percentile rank, reflecting the ability of a specific peptide to bind to a particular HLA molecule and elicit a potent immune response.

For neo-antigen prediction, FASTA sequences of the E6 and E7 proteins were accessed from the NCBI Database. The HLA class I and class II dataset recommended by the IEDB were used. For the selection of strong class I binders, we used the IEDB recommendations of an IC_50_ below 50 nM, and a percentile rank < 2%. In the case of class II strong binders, an IC_50_ < 50 nM, along with a percentile rank < 10%, were considered.

### 2.3. Allergenicity and Toxicity in In Silico Screening

As peptides may trigger an IgE-mediated hypersensitivity reaction which may manifest clinically from skin rash and pruritus to severe anaphylactic reactions, allergenicity prediction is an important selection process for peptide design. In addition, toxicity screening is mandatory, given the fact that many animal venoms contain peptides with potent neuro- and hematotoxic activity. To prevent unwanted hypersensitivity or toxic reactions, peptides were screened in silico for allergenicity and toxicity.

The allergenicity prediction assay was performed using AllerCatPro v.2.0, a web-based algorithm that identifies, for a given FASTA sequence, both linear and discontinuous epitopes with allergenic potential through a hexamer hit screening, a gluten-like pattern recognition, and a 3D structure comparison with 4180 already known allergenic proteins [19].

ToxIBTL is a hybrid deep-learning model that classifies both short and long amino acid sequences. The screening algorithm involves peptide sequence encoding as a BLOSUM62 scoring matrix. Then, the evolutionary information from the BLOSUM62 matrix is inputted into a 2D convolutional neural network that uses the ReLU non-linear activation function to extract correlations between amino acids [20].

To extract features from protein sequences, the FEGS model is used. FEGS extracts graphical and statistical features of peptide sequences based on 158 physicochemical properties of amino acids. Hence, each peptide is transformed into a 158-dimensional numerical vector. For each property, the amino acids are represented graphically on a right circular cone with a height of 1. Then, a 3D graphical curve of the peptide is constructed. The corresponding 2D non-symmetrical matrix for a given physicochemical property is constructed and its largest eigenvalue is determined [21].

Combining statistical features, such as amino acid and dipeptide composition, the vectors are refined, using the information bottleneck principle, and fed to a ReLU activation function layer and a sigmoid. The result shows the probability that a given peptide/protein is toxic. The values range from 0 to 1 and a score > 0.5 reflects toxicity [20].

### 2.4. Population Coverage Analysis

Optimal peptide vaccine production requires careful analysis of the most frequent HLA alleles that bind the peptide set. Peptides bind with various affinities to specific human leukocyte antigen (HLA) molecules. By knowing the affinity of each peptide to certain HLA molecules and the allele frequency in the world population, the population coverage of a given peptide set can be computed. The IEDB population coverage analysis tool inputs peptides with specific HLA-binding repertoire and calculates the percentage of a population of interest that is covered by the peptide pool [22]. The HLA class I and class II allele reference dataset was used for population coverage analysis.

### 2.5. Peptide Selection Criteria

The main criteria for epitope selection included a high antigenicity score, high promiscuity (binding to a larger number of HLA alleles), non-toxicity, non-allergenicity, and favorable physicochemical properties.

### 2.6. Design of Synthetic Long Peptide Construct Linker Selection

Our proposed vaccine candidates comprise two subunits joined by the designed cleavable linker, LRMK. Each subunit consists of an N-terminal class II-restricted epitope, the cathepsin-sensitive linker LLSVGG, and a class I-restricted antigenic sequence at the C terminus.

The rationale for this construct is based on the following hypotheses:

1. HLA class II molecules are less restrictive in peptide size and may bind larger epitopes, including linker fragments.
2. Endoplasmic reticulum aminopeptidases (ERAPs) cleave the remaining class I epitope bound to the linker fragment, therefore fitting the class I-restricted molecule inside the HLA class I-binding pocket.
3. Rabu et al. described a 100-fold increase in antigen presentation and cross-presentation of synthetic long peptides by dendritic cells by using the LLSVGG cathepsin-sensitive linker, compared to other linkers (LVGS, LLSV, GGGG, etc.) [23].
4. ERAP1 has a higher specificity for hydrophobic amino acids such as leucine and methionine, while ERAP2 cleaves basic residues such as lysine or arginine [24].
5. The LRMK linker is also a good substrate for cathepsin S, enhancing cross-presentation and providing protective cellular immunity against overlapping peptide proteins [25].

### 2.7. Physicochemical Properties of Synthetic Long Peptides

Peptide candidates were characterized based on their physicochemical properties using the ProtParam library for BioPython.

Guruprasad et al. demonstrated, based on a statistical analysis performed on 32 stable and 12 unstable proteins, that the instability of a polypeptide chain correlates with the presence of specific dipeptides. From these observations, the authors described the instability index (II) which correlates with poor stability in vivo when greater than 40 [26].

The Grand Average of Hydropathy (GRAVY) was computed by the addition of hydropathy values, determined by Kyte and Doolittle [27], of all amino acids contained in each SLP and divided by the total number of residues. A positive GRAVY value is correlated with an overall hydrophobic polypeptide, whereas a negative value suggests hydrophilicity [28].

### 2.8. Antigenicity Assay Using VaxiJen 2.0

Vaxijen 2.0 is the first alignment-independent antigenicity prediction based exclusively on the physicochemical properties of a given peptide/protein. The prediction method relies on auto-cross-covariance (ACC) transformation of amino acid sequences into vectors of equal length containing the principal z-descriptors. The z-descriptors of amino acid sequences were established by Hellberg et al., based on the principal component analysis (PCA) of 29 amino acid physicochemical properties and dependent upon hydrophobicity (z1), geometric features (z2), and polarity (z3) [29]. The output of VaxiJen is a score that reflects the probability of an amino acid sequence to be antigenic. A VaxiJen score greater than 0.4 (the viral antigen threshold) reflects probable antigenicity [30].

### 2.9. 3D Structure Prediction

Three-dimensional structure prediction was performed using the Rosetta ab initio protocol. For each synthetic long peptide, 3-mer and 9-mer fragment libraries were generated using Robetta (http://old.robetta.org/fragmentsubmit.jsp, accessed on 1 February 2023), by searching in the Protein Data Bank (PDB) for all known conformations that a 3-mer or 9-mer can adopt inside an already solved protein structure. Fragment assembly generates models with different conformations based on physicochemical interactions between amino acid residues, and on the probabilities that specific backbone orientations are conserved throughout evolution. The physico-chemical parameters, along with the statistical terms, are used by the Rosetta scoring function, ref2015, to attribute to each synthetic long peptide a Rosetta score [31].

For each SLP structure, we generated 10,000 decoys, which were clustered based on their Rosetta score and root mean square deviation (RMSD) from the initial conformation. Because E6 and E7 proteins from HPV-16 and HPV-18 are already solved and stored inside PDB, the initial conformation for each SLP was determined by homology modeling, using SwissProt. From the best cluster (lowest RMSD and lowest Rosetta score) the model with the lowest Rosetta score was selected for further analysis.

### 2.10. 3D structure Validation QMEAN Score Ramachandran Plots

Overall stereochemical quality assessments of the generated models was performed using PROCHECK, and the corresponding Ramachandran plots were drawn. Global quality analysis was computed using SWISS-MODEL’s Quality Model Energy Analysis (QMEAN) score. The QMEAN scoring function is a weighted sum of structural descriptors which describe the local molecular geometry (3-mer torsion potentials), long-range interactions (Cβ-all atom interactions), solvation potential, and solvent accessibility. QMEAN scores range from 0 to 1, with 0 representing a poor-quality model, and 1 representing a high-quality model. Additionally, the Z-score reflects the QMEAN score comparison between the input sequence and a non-redundant set of NMR or X-ray crystallography-solved PDB structures. A Z-score value closer to 0 suggests that the analyzed polypeptide chain possesses fragments with similar conformations to native protein structures, while a Z-score below −4 indicates a low-quality model [32].

A good-quality model was considered to have > 90% of the total residues in the allowed regions, with no aberrant values of the φ and ψ angles, a > 0.5 QMEAN4 score, and a QMEAN Z-score above −2.0.

### 2.11. Molecular Docking Studies with Toll-Like Receptor 2

Besides adaptive immunity, an investigation of potential innate immune system activation by synthetic long peptides is required. 

Toll-like receptor 2 (TLR2) is a membrane-bound protein that recognizes pathogen-associated molecular patterns (PAMPs) and triggers inflammatory cytokine production and release. In addition, it activates the dendritic cells and improves antigen presentation. Its biochemical structure comprises an extracellular domain, containing 10–30 leucine-rich repeats that recognize PAMPs, a transmembrane region, and a cytoplasmic domain, which plays an important role in signal transduction and subsequent inflammatory cytokine release [33].

To assess the capacity of SLPs to bind TLR2, molecular docking studies using HADDOCK were performed.

HADDOCK 2.4 is a molecular docking script collection that accesses the Crystallography and NMR System (CNS) experimental library, along with geometric and energy-based calculations for guiding the docking process. 

The HADDOCK protocol begins with the identification of the most geometrically favorable binding surfaces between the ligand (SLP) and the receptor (TLR2). During this step (it0), both the ligand and the receptor are treated as rigid objects. HADDOCK generates 1000 models in this step, but only the top 200 decoys are kept for further analysis. The next step is a three-step molecular dynamics-based flexible docking protocol, in which the torsion angles between the interacting residues are adjusted to maximize the number of strong intermolecular bonds (ionic interactions, hydrogen bonds). The last step is an energy minimization protocol consisting of a short molecular dynamic simulation at 300 K in a box of water molecules (TIP3P model). The latter step further adjusts the torsion angles to minimize the area accessible for solvent molecules [34,35].

### 2.12. Binding Affinity Calculation Using the PRODIGY Webserver

For binding energy and dissociation constant calculation, we used the PRODIGY webserver. PRODIGY (PROtein binDIng enerGY prediction) uses a linear regression model based on the number of interfacial contacts (polar/apolar/charged) between the receptor-ligand (TLR2-SLP) interacting residues. Two residues are considered in contact if the distance between them is <5.5 Å [36,37].

Starting from the binding affinity (ΔG), the dissociation constant (K_d_) can be calculated:

$$\Delta G=RT\ln K_{d}$$

where R—ideal gas constant (0.082 kcal × K^−1^ × mol^−1^), T—temperature (K).

### 2.13. Molecular Dynamics (MD) Simulations

To better understand the interactions between the peptide and the solvent, as well as the stability and dynamics of the peptide over time, MD simulations were performed using the GROMACS 2023.1 package.

The selected force field was OPLS-AA. Each SLP was solvated using the SPC/E water model in a cubic box with a volume of 300 nm^3^ and centered. Electrical neutralization of the system was performed by adding Na^+^ and Cl^−^ ions. Each simulation started with energy minimization for 5000 steps, using the steepest descent method, which involved finding the minimum energy configuration of the system by iteratively adjusting the positions of the atoms until the forces acting on them were minimized [38].

After minimization, three MD simulation steps were performed: NVT, NPT, and MD production run.

NVT (constant number of particles, constant volume, constant temperature) equilibration was performed using the v-rescaled Berendsen thermostat [39], while the NPT (constant number of particles, constant pressure, constant temperature) equilibration was done with the Parinello-Rahman barostat [40]. Both MD simulations were performed for 100 ps. The electrostatic interactions were computed using the particle-mesh Ewald (PME) method [41] and the linear constraint solver (LINCs) [42].

Finally, a full molecular dynamics simulation of the system of 100 nanoseconds (ns) with periodic boundary conditions was performed. This allowed the simulation to capture the behavior of the peptide in a solvent environment (water) over an extended period. Each MD production simulation was performed at 300 K for 100 ns, with a time step equal to 2 fs (0.002 ps). The MD trajectories were analyzed using gmxrms (for root mean square deviation, RMSD), gyrate (for radius of gyration, Rg), gmxrmsf (for root mean square fluctuation, RMSF), and gmx sasa packages, which provided information about the stability and conformation of the peptides over time.

The root mean square deviation (RMSD) quantifies the differences between the initial and simulated conformations of a molecule over time. The RMSD is calculated as the root mean square of the distances between backbone corresponding atoms in the two structures. These distances are calculated after the two structures have been superimposed, so that their centers of mass are aligned:

$$RMSD\;\left(a,\;b\right)=\sqrt{\frac{1}{n}\overset{n}{\underset{i=1}{\sum }}\left[{\left(a_{ix}-b_{ix}\right)}^{2}+{\left(a_{iy}-b_{iy}\right)}^{2}+{\left(a_{iz}-b_{iz}\right)}^{2}\right]}$$

where n—total number of atoms, a_ix_, a_iy_, a_iz_, b_ix_, b_iy_, b_iz_—coordinates of atom i from the initial conformation and the simulated conformation b on the x, y, and z-axes;

A lower RMSD value indicates that the simulated structure closely resembles the experimental structure, while a higher RMSD value suggests a significant conformational change. RMSD dynamics were also evaluated, with subtle changes over time reflecting high stability.

The root mean square fluctuation (RMSF) evaluates the flexibility of an amino acid residue i by determining its deviation from the average position of the residue over the course of the MD simulation:

$$RMSF_{i}=\sqrt{\frac{1}{T}\overset{T}{\underset{t=1}{\sum }}{\left(r_{i}\left(t\right)-r_{i}^{ref}\right)}^{2}}$$

where T—duration of MD simulation, t—timestep, r_i_(t)—coordinates of atom i at timestep t, r_i_^ref^—coordinates of atom i in the initial (reference) conformation.

The RMSF was used to identify the most flexible and rigid regions of a molecule. A high RMSF value for a residue indicates high mobility and flexibility, while a low RMSF value indicates a relatively rigid amino acid.

The radius of gyration (R_gyr_) is defined as the root mean square distance of the atoms from the molecular center of mass:

$$R_{gyr}=\sqrt{\frac{1}{M}\overset{N}{\underset{i=1}{\sum }}m_{i}{\left(r_{i}-R\right)}^{2}}$$

where R_gyr_—radius of gyration, M—total mass of the molecule, R—the center of mass for the molecule, m_i_—mass of the atom i, r_i_—distance of atom i from the origin of the cartesian system.

Therefore, R_gyr_ reflects the size, shape, flexibility, and stability of a molecule in molecular dynamics simulations.

A smaller R_gyr_ indicates that the atoms in the molecule are more compactly distributed, while a larger value suggests a looser conformation.

The radius of gyration was calculated at different points in time to track changes in the size and shape of the molecule as the simulation progresses. This information can be used to study the dynamics of the molecule and to understand how its structure is affected by changes in temperature or pressure.

## 3. Results

### 3.1. Class I- and Class II-Restricted Epitope Identification

One hundred and forty-nine unique class I (43 epitopes, 28.85%) and class II (106 epitopes, 71.15%) epitopes were identified from HPV-16 E6 and E7 proteins, based on the search criteria. Of the 26 class I epitopes that expressed a percentile rank < 0.5% (Table 1), 7 epitopes expressed the highest promiscuity (could bind more than 2 HLA class I alleles). To enhance the recognition repertoire and achieve optimal population coverage, 5 additional epitopes were predicted from E6 and E7 FASTA sequences (accessed via NCBI), using the IEDB class I consensus prediction algorithm. 

Out of the 17 identified class II epitopes, six of them expressed a percentile rank < 10%, bound more than 2 HLA class II alleles, and provided adequate population coverage (94.8%) (Table 2).

For HPV-18 E6 and E7 proteins, we have identified 5 class I-restricted antigenic sequences from the IEDB database, along with 10 class I-predicted epitopes, which could provide 79.8% coverage for the world population. Three IEDB and 8 predicted class II epitopes cover 98.18% population coverage for the most frequent HLA alleles in the world.

### 3.2. Population Coverage Analysis

Population coverage analysis for the combined HPV-16 and HPV-18 class I dataset provided a world population coverage of 98.18%, an average hit of 6.48, and a PC90 of 2.47, suggesting that 90% of the world population will recognize a minimum of 2 epitopes from this dataset.

The corresponding class II coverage parameter output highlighted 99.81% world coverage, an average hit of 15.29, and a minimum number of 9 epitopes that would be recognized by 90% of the population (Table 3).

### 3.3. SLP Construct Design

SLPs were constructed by linking 2 antigenic subunits with the ERAP- and cathepsin S-sensitive cleavable linker, LRMK. Each antigenic subunit consists of a class II-restricted epitope located at the N-terminus, the cathepsin-sensitive linker LLSVGG, and a class I-restricted epitope at the C-terminus. Every construct provides 4 antigenic sequences that can be canonically and cross-presented to CD4+ and CD8+ T cells.

Out of the total possible constructs (>200,000 combinations), physicochemical property analysis and antigenicity screening provided 25,000 structures, with an instability index below 40 and a VaxiJen score above 0.4.

The additional selection process identified 25 constructs, with the maximum VaxiJen score, minimum instability index, and maximum diversity (maximum number of different epitopes included in one construct). (Table 4)

### 3.4. Physicochemical Property Analysis

Physicochemical property analysis reveals that the 25 synthetic long peptides have a molecular weight of 7.5 ± 0.3 kDa, express high antigenicity (mean VaxiJen score = 0.97), are stable under in vitro conditions (mean instability index = 37.61), and are slightly hydrophobic (mean GRAVY score = 0.38). Based on their N-terminal amino acids, the 25 SLPs have a half-life of 15.76 ± 13.03 h inside a mammalian cell.

### 3.5. In Silico Toxicity and Allergenicity Assay

Toxicity prediction revealed that all 25 SLPs express a close-to-zero ToxIBTL probability (mean ToxIBTL score = 1.5 × 10^−2^), which translated into a low likelihood for toxic adverse effects. Allergenicity in silico screening provided negative results for triggering hypersensitivity reactions.

### 3.6. Three-Dimensional Structure Prediction

For each SLP, we constructed a fragment library consisting of 3-mers and 9-mers with known secondary conformation. Using Rosetta ab initio, we predicted 10,000 decoys for each antigenic construct that were further clustered based on their Rosetta score and RMSD. The models with the lowest Rosetta score, RMSD, and the best 3D structure analysis parameters were selected for further analysis.

### 3.7. Three-Dimensional Structure Validation

**Figure 1:**
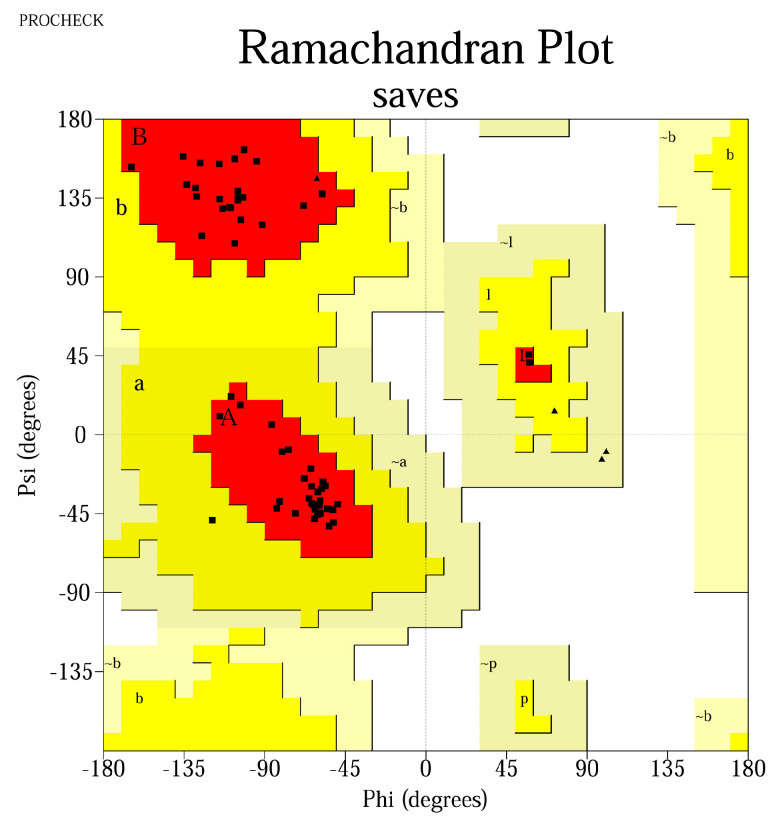
Ramachandran plot for the peptide MLDLQPETTDLYCYELLSVGGKFYSKISEYLRMKLKFYSKISEYRHYCYLLSVGGLFLNTLSFV. Most residues are located in the most favored regions (98.3%), while 1.7% are in the additional allowed regions, confirming a high−quality model. The Gly residues are represented as triangles, whereas the squares represent the other amino acids. The red areas represent the core region in which the most favorable combinations of φ-ψ angles are located (defined as A, B, P, L); a, b, p, l represent the additional allowed regions (yellow areas), while −a, −b, −l, −p are the generously allowed regions (light yellow areas).

The QMEAN4 score was between [0.665; 0.813], while the Z-score was between [−2.03; 0.3], implying that the designed models express similarities with structures already found in nature. PROCHECK analysis identified that the residues were located >90% in most favorable regions, suggestive for thermodynamically stable conformations (Table 5). The results were represented graphically using Ramachandran plots (Figure 1).

**Figure 2:**
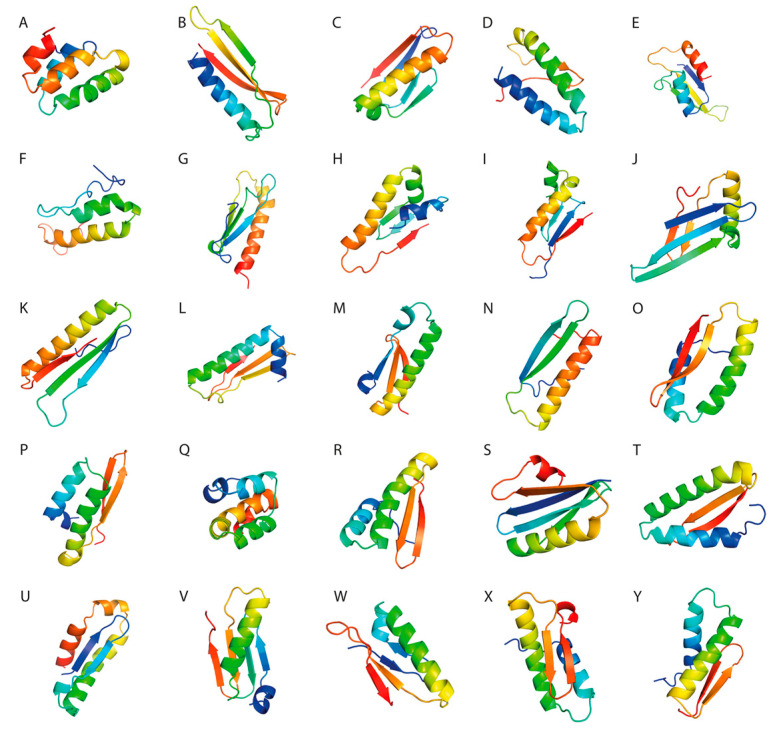
(**A**–**Y**). Molecular visualization of the peptide constructs using PyMol software.

Three-dimensional structure visualization was performed using PyMol software (Figure 2).

### 3.8. Molecular Docking Studies of the TLR2-SLP Complexes

Molecular docking analyses provided a HADDOCK score of −151 ± 18.77. We found that the electrostatic energy had the biggest contribution to the TLR2-SLP interactions. Further statistical analysis has shown that electrostatic energies make a notably greater contribution than the van der Waals interactions (*p* < 0.001). The desolvation energies had a predominantly negative value, suggesting that water dissociates freely from the interacting surfaces, allowing receptor-ligand interaction. The restraints violation energy values were close to 0 (mean E_AIR_ = 0.852), suggestive for good-quality docking simulations (Table 6).

### 3.9. Free Energy Determination

Gibbs free energy and dissociation constants for the TLR2-SLP complexes were calculated using the PRODIGY webserver. The results showed a ΔG of −13.20 ± 1.42 kcal/mol and an average K_d_ of 3.5 × 10^−9^ M, suggestive of a thermodynamically stable interaction between the designed constructs and the toll-like receptor 2 (Table 7).

**Figure 3:**
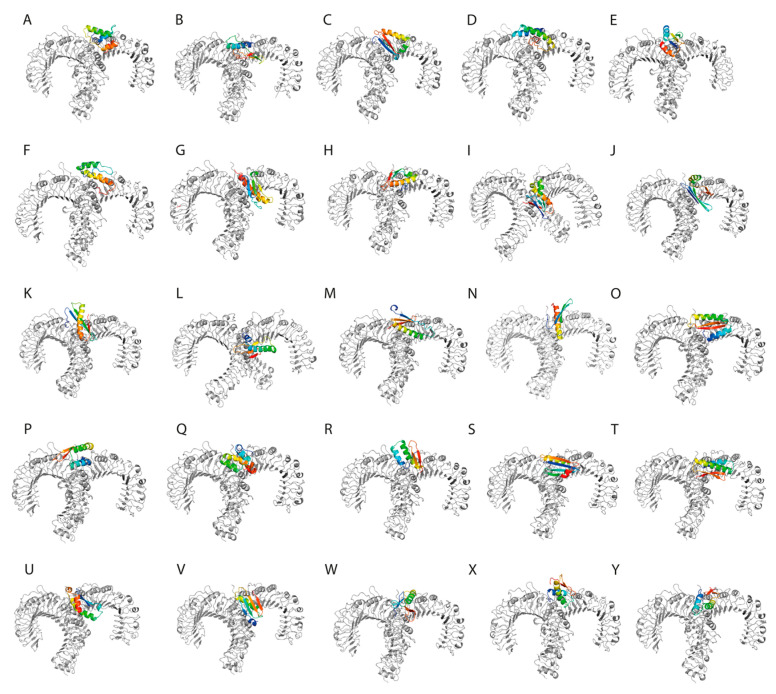
(**A**–**Y**). The designed synthetic long peptides in complex with toll-like receptor 2.

The interaction between TLR2 and the SLPs was visualized using PyMol (Figure 3).

### 3.10. Molecular Dynamics Simulations

**Figure 4:**
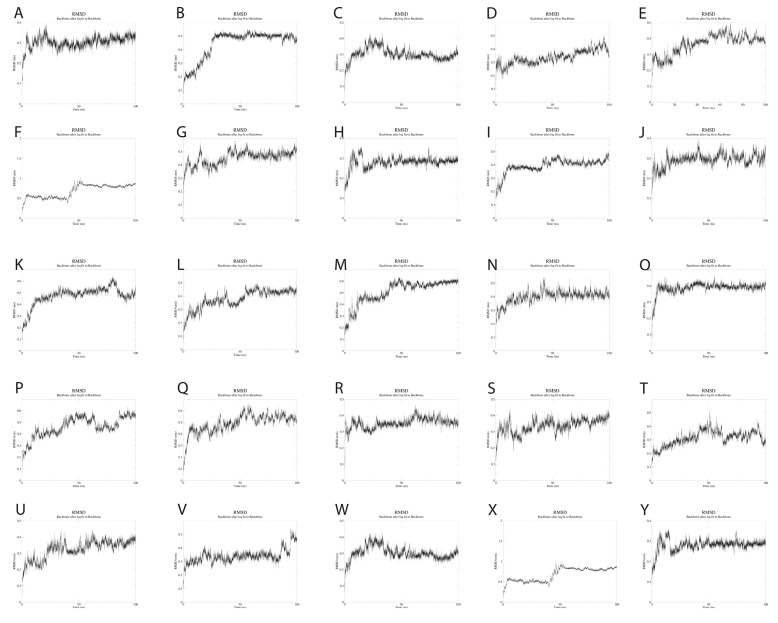
(**A**–**Y**). RMSD changes during the 100 ns simulations of the 25 SLPs. All SLPs adopt a stable conformation during the MD simulation. Peptides 6 and 24 change their conformation at 40 ns simulation, but the newly adopted conformation is maintained during the 40–100 ns simulation time.

To evaluate the dynamics of the synthetic long peptide constructs, we performed molecular dynamics simulations. The RMSD of the backbone, RMSF, and radius of gyration were analyzed for 100 ns. Backbone RMSD values ranged between [0.1; 0.87] nm, with mild fluctuations over the 100 ns simulation time, suggesting increased construct stability during simulation. Peptides 6 and 24 expressed an RMSD shift at 40-ns, but with mild fluctuations over the 40–100 ns time interval (Figure 4).

The Rg values showed very little fluctuation throughout the simulation, with the average Rg being 1.22 nm. The lowest Rg value observed was 1.15 nm, while the highest was 1.34 nm. These results reflect the compactness of the SLP constructs, with increased stability over time. 

**Figure 5:**
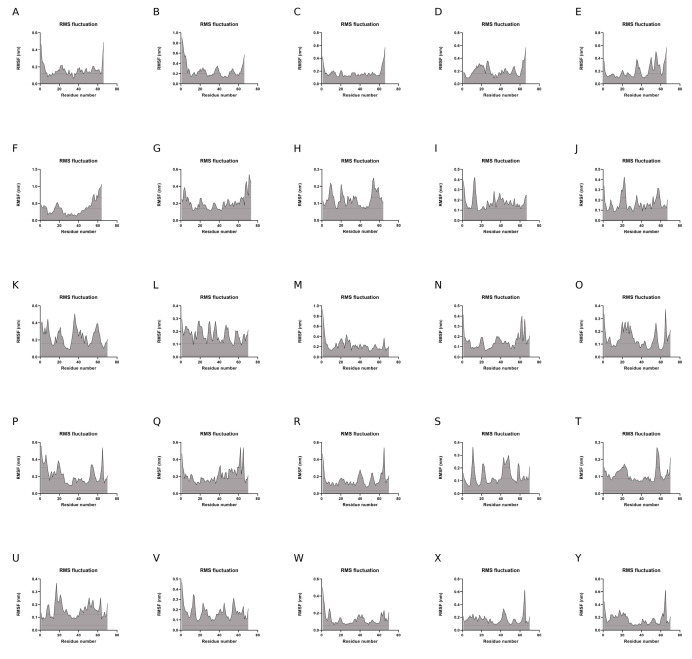
(**A**–**Y**). RMSF plots of Cα atoms during the 100-ns simulations.

**Figure 6:**
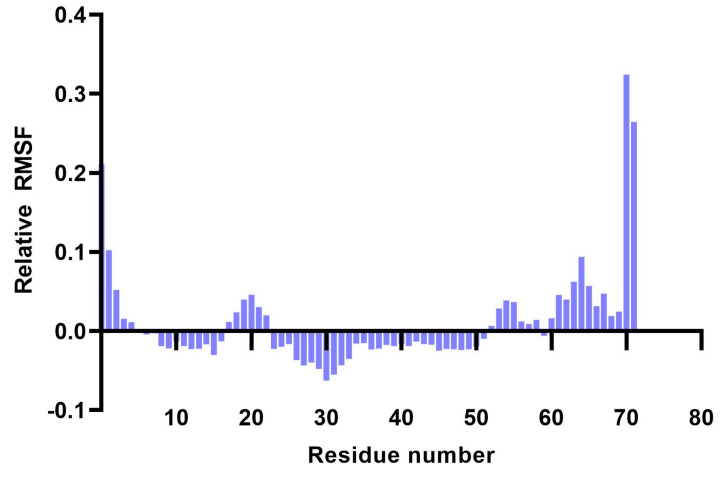
Plot showing the variation of residue flexibility as a function of residue number. The relative RMSF was computed with respect to the mean RMSF for each residue number. It is worth noting that residues 30–35 express the lowest flexibility (corresponding to the LRMK linker), while residues 15–25 and 51–57 express the highest flexibility (corresponding to the LLSVGG linkers).

Analysis of RMSF plots showed that the residues present in positions 15–25 and 51–57, corresponding to the LLSVGG linkers, express the highest flexibility. Conversely, residues located in positions 30–35, corresponding to the LRMK linker, expressed the lowest flexibility (Figure 5 and Figure 6).

**Figure 7:**
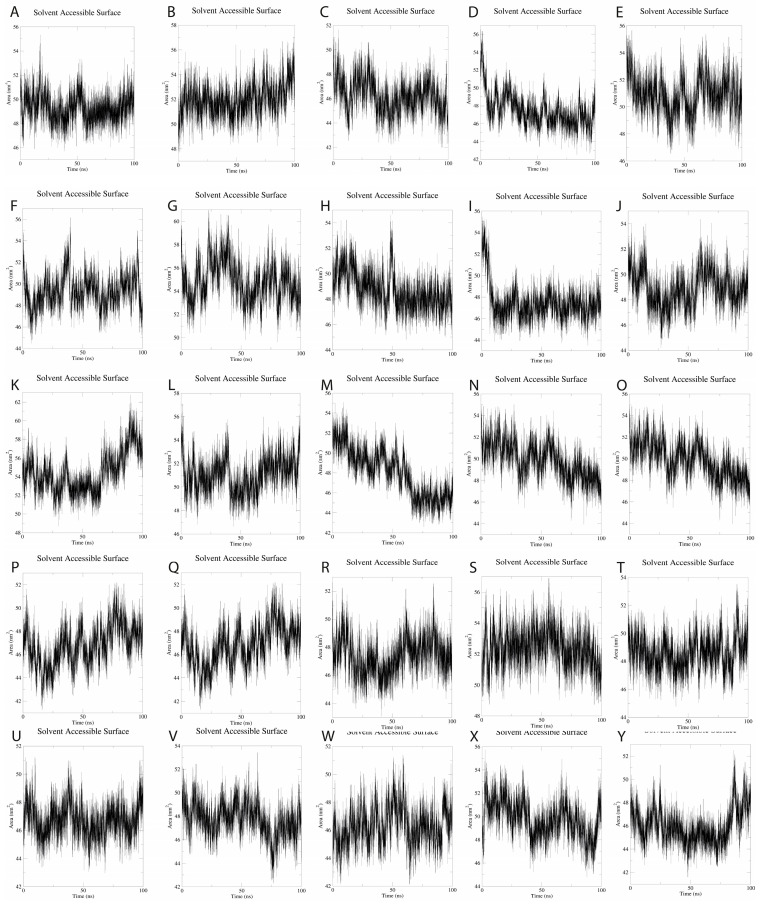
(**A**–**Y**). SASA plots for the 25 SLPs present an average SASA of 49.37 nm^2^, with fluctuations of 1.54 nm^2^ during the 100-ns simulations.

SASA (Solvent Accessibility Surface Area) analysis over the 100-ns simulation time period provided a 49.37 ± 1.54 nm^2^, suggesting mild fluctuations during MD simulations. These findings suggest that the 25 SLPs adopt a stable conformation when dissolved in water (Figure 7).

## 4. Discussion

HPV-16 and -18 infection represents a significant public health issue, due to its high oncogenic potential. Despite the growing availability of prophylactic vaccines, HPV-related neoplasia remains the fourth most common cancer worldwide.

The three VLP-based vaccines induce antibody production, thereby inhibiting HPV-keratinocyte interaction. However, there is no effect on already HPV-infected or malignantly transformed cells. This finding is attributed to L1 antigen downregulation.

E6 and E7 are the main HPV proteins that drive malignant transformation by enhancing the degradation of p53 and pRb tumor suppressor proteins. Because of this, along with their high conservancy among the HPV subtypes, epitopes originating from these two proteins were used in this vaccine design.

Peptide-based vaccination represents a promising alternative to classic vaccination platforms. Even though peptides alone possess a low immunogenic potential, adjuvants or immunostimulatory molecules can be added to elicit proper dendritic cell and T cell stimulation.

HPV-related cancers proliferate due to poor CD4^+^-mediated cytokine secretion, CD8^+^-associated cytotoxicity, and tumor penetrability, along with a high influx of regulatory CD4^+^ FOXP_3_^+^ T cells [64].

The intended role of our proposed vaccine platform is to clear the infected cells before the malignant transformation occurs. Therefore, this design should exert both a therapeutic (pre-malignant cell clearance) and prophylactic effect (carcinoma prevention).

Several clinical trials that utilized HPV-derived SLPs were conducted. In a study performed by Welters et al., synthetic long peptides comprised of overlapping E6 and E7 peptides were administered to HPV-16+ cancer patients. All patients displayed an increase in blood HPV-16-specific CD4+ and CD8+ cells, with proportional interferon-γ release, which are all hallmarks for a rebalancing anti-tumoral immune response [65]. Van Poelgeest et al. performed a phase I clinical trial on women with HPV-16+ gynecological carcinoma, who were immunized subcutaneously with HPV-16 E6 and E7-overlapping long peptides. The results have shown no systemic toxicity, but a vaccine-induced anti-tumor response with increased IFN-γ, TNF-α, IL-5 and IL-10 production. Although the vaccine was well tolerated and provided enhanced cytokine secretion, the investigators did not observe any tumor regression or halting of the malignant process [66]. Speetjens et al. conducted a phase I vaccination study on patients with HPV-related carcinomas using HPV-16 SLPs conjugated with the TLR2 agonist, Amplivant. The results revealed a dose-dependent T cell response with subsequent cytokine release (IFN-γ, IL-5). It was also observed that the concentration of pro-inflammatory cytokines increased significantly when the vaccine was administered in combination with chemotherapy [65,67]. Although various pre-clinical and clinical studies have shown an increased number of HPV-specific CD4+ and CD8+ T cells after immunization, with proportional cytokine secretion, the activity of tumor-therapeutic vaccines against established tumors is limited [65,66,67,68,69]. One possible explanation involves the immunosuppressive behavior of the tumor microenvironment against the vaccine-stimulated T cells, which stresses the need for cancer prophylaxis.

Compared to the aforementioned studies which used overlapping peptides, our proposed design consists of isolated peptides that were either already identified as strong immune enhancers or predicted in silico. The proposed design includes epitopes from various regions of the whole protein sequences, thereby priming the T cells against multiple antigenic regions. By including the flexible, cleavable linker LLSVGG, both canonical and cross-presentation are enhanced 100-fold, based on the in vitro and in vivo studies performed by Rabu et al [23]. Furthermore, the more rigid, cleavable linker LRMK used in our constructs was also used in combination with HPV-16 E7 recombinant peptides in a HPV-16 E7-B16 melanoma murine model. The results display both an increased CD8+ and CD4+ T cell immune response against E7-expressing melanoma B16 cells, with increased survival compared to the control group [25].

Linker usage provides a highly specific proteolysis compared to the overlapping peptide designs, in which cleavage may occur randomly.

Twenty-six class I-, and 18 class II-, restricted epitopes were either selected from the Immune Epitope Database or predicted using artificial neural networks. The peptides with the highest promiscuity and highest binding affinity to HLA molecules were selected.

This particular vaccine design was chosen to achieve an increased immune response against the virally infected cells in the global population. Multiple epitopes from E6 and E7 proteins of HPV-16 and HPV-18 were selected: 26 HLA class I-restricted, and 18 class II-restricted. Given the low prevalence of other genotypes in cervical carcinomas [6], including another set of 10–15 epitopes was considered impractical.

According to population coverage analysis, it is estimated that there is a 90% likelihood that individuals possessing an HLA allele listed in the IEDB database can identify at least 2 class I peptides and 9 class II epitopes. The analysis revealed that the combined class I coverage was 98.18%, and the class II coverage was 99.81%. It is important to note that further clinical studies are needed to fully understand the peptide recognition repertoire. The cellular immune response is specific to each individual, based on the particular HLA class I and HLA class II haplotypes. Population coverage requires selecting a combination of peptides, which can interact with the various HLA haplotypes present in the population. Vaccines that are built using only a small number of peptides may fail in the population, being active only in a small proportion of individuals. Our strategy overcomes this by employing a set of peptides that interacts with the HLA molecules present in the majority of the population.

The design of peptide-based vaccines requires screening for potential allergenic or toxic reactions. In silico studies allow a rapid and cost-effective screening process. Allergenicity analysis using AllerCatPro revealed that none of the 44 peptides or 25 synthetic long peptides possess allergenic potential, while toxicity analysis using ToxiBTL provided low probability for toxic adverse effects. However, the results need to be further validated through in vitro and in vivo studies.

Combining class I- and class II-restricted epitopes achieves a bidirectional stimulation of both cytotoxic and helper T cells. The class I epitopes provide the main immunogenic target for CTLs, while class II epitopes trigger cytokine release, further augmenting the immune response.

The 25 synthetic long peptides’ pool presents a considerable degree of redundancy, which might be useful when performing experimental validation and reducing the risk of loss of the initial class I- and class II-restricted epitopes during non-specific cleavage.

Ab initio three-dimensional structure prediction using Rosetta provided good-quality models, with >90% of the residues located in the most favorable regions on the Ramachandran plot, as well as QMEAN4 scores > 0.7, which suggest that the 3D-modeled decoys adopt conformations similar to other structures found in nature.

Toll-like receptors (TLRs) play a crucial role in the innate immune response, maturation of dendritic cells, and enhancement of antigen presentation. Besides cytokine secretion and upregulation of co-stimulatory molecules, toll-like receptors (especially toll-like receptor 2) also play an important role in antigenic cross-presentation [68]. De Vos van Steenwijk et al. showed that ex vivo stimulation of T cells from infiltrated cervical cancers and sentinel lymph nodes with HPV-16 E6 and E7 peptides, in combination with TLR agonists such as lipopolysaccharide or Pam3CSK4, increased IFN-γ production, suggesting that tumor-infiltrated lymphocytes (TILs) and tumor-draining lymph node cells (TDNCs) are present in high numbers in HPV-related tumors, but are suppressed by the tumor microenvironment [69].

The designed synthetic long peptides expressed a good binding affinity to toll-like receptor 2, supported by negative Gibbs free energy and low dissociation constants. These results suggest that SLPs possess intrinsic TLR2-agonist activity, which may further reduce the need for adjuvants.

Molecular dynamics simulations of the 25 SLPs revealed that the designed peptides during the 100 ns simulations adopt a stable and compact conformation when dissolved in water. Analysis of RMSF plots show that the residues present in the positions 15–25 and 51–57, corresponding to the LLSVGG linkers, express the highest flexibility. These findings are supported by experiments conducted by de Bold et al. [70] and Waldo et al. [71], who reported that flexible linkers are generally rich in small or polar amino acids, such as serine or glycine. Higher flexibility allows mobility between the antigenic components and favors TLR and HLA interactions. Residues located in positions 30–35, corresponding to the LRMK linker, expressed the lowest flexibility. Therefore, LRMK acts as a spacer between the 2 antigenic subunits, while maintaining the overall stability of the SLP. Kallinteris et al. showed that linking the LRMK linker to MHC class II epitopes enhanced antigen presentation, both in vitro and in vivo. The suggested mechanism involves the binding of LRMK to an allosteric site on the MHC class II molecule, with a subsequent increase in epitope loading [72].

Despite the positive in silico results, the present study has some limitations. The main limitation of this study is the lack of experimental validation. To fully characterize the immunogenic, allergenic, and toxic potential for the 25 SLP set, both in vitro and in vivo validation needs to be performed. Another limitation is that immunoinformatic methods cannot predict factors that influence vaccine uptake, delivery, and host immune response, but further clinical studies are able to answer the unsolved questions. The last major limitation of the current vaccine is the requirement for a functional cellular immune response. Patients with an impaired immune system may develop only a poor response to this vaccine. Women with HIV infection have an increased risk to develop cervical carcinoma; thus, the vaccine may fail to clear the HPV infection in this population group. This limitation may affect all such vaccines.

## Figures and Tables

**Table 1:** Class I-restricted epitopes.

Epitope	Source	Alleles	Protein	HPV Type
YMLDLQPETT	Literature ([43,44,45])	HLA-A*02:01	E7	16
TIHDIILECV	Literature ([46,47,48,49])	HLA-A*02:01, HLA-B*07:02	E6	16
YAVCDKCLKF	Literature ([50])	HLA-A*24:02, HLA-B*35:01	E6	16
EYRHYCYSL	Literature ([51,52])	HLA-A*02:01, HLA-B*07:02, HLA-A*24:02, HLA-B*35:01	E6	16
FAFSDLYVVY	Literature ([53])	HLA-B*35:01, HLA-A*26:01, HLA-A*24:01	E6	18
KLPDLCTEL	Literature ([54,55,56])	HLA-A*02:01, HLA-A*02:03	E6	18
LFLNTLSFV	Literature ([54])	HLA-A*02:01	E7	18
SVYGDTLEK	Literature ([57])	HLA-A*11:01	E6	18
HTMLCMCCK	Literature ([57])	HLA-A*11:01, HLA-A*30:01	E7	18
KFYSKISEY	Prediction	HLA-A*30:02, HLA-B*15:01, HLA-A*30:01, HLA-A*32:01, HLA-A*23:01	E6	16
CPEEKQRHL	Prediction	HLA-B*07:02, HLA-B*08:01, HLA-B*53:01	E6	16
YGTTLEQQY	Prediction	HLA-B*35:01, HLA-A*30:02, HLA-A*01:01	E6	16
TTLEQQYNK	Prediction	HLA-A*11:01, HLA-A*68:01, HLA-A*03:01	E6	16
RAHYNIVTF	Prediction	HLA-B*57:01, HLA-B*58:01, HLA-B*15:01, HLA-A*32:01, HLA-B*35:01, HLA-B*53:01, HLA-A*23:01	E7	16
LQPETTDLY	Prediction	HLA-B*15:01, HLA-A*30:02, HLA-A*01:01	E7	16
TPTLHEYML	Prediction	HLA-B*07:02, HLA-B*53:01, HLA-B*35:01, HLA-B*08:01, HLA-B*51:01	E7	16
FAFKDLFVV	Prediction	HLA-A*02:06, HLA-A*02:01	E6	18
LQDIEITCVY	Prediction	HLA-B*15:01	E6	18
LIRCLRCQK	Prediction	HLA-A*30:01	E6	18
FYSRIRELR	Prediction	HLA-A*33:01	E6	18
FAFKDLFVVYR	Prediction	HLA-A*68:01	E6	18
ELTEVFEFA	Prediction	HLA-A*68:02	E6	18
RFHNIAGHY	Prediction	HLA-A*30:02	E6	18
FEFAFKDLF	Prediction	HLA-B*40:01	E6	18
RFHNIAGHYR	Prediction	HLA-A*31:01	E6	18
LNTLSFVCPW	Prediction	HLA-B*57:01, HLA-B*58:01	E7	18

**Table 2:** Class II-restricted epitopes.

Epitope	Source	HLA Alleles
CVYCKTVLELTEVPAV	Literature ([58])	HLA-DPA1*02:01, HLA-DPB1*01:01,
LFMDSLNFVCPWC	Literature ([59])	HLA-DRB3*01:01,
DLFVVYRDSIPHAACHKCIDFY	Literature ([58])	HLA-DRB1*03:01
MHGDTPTLHEYM	Literature ([60,61,62])	HLA-DQB1*02:01
DRAHYNIVTFCCKCD	Literature ([59])	HLA-DRB1*15:01
DSTLRLCVQSTHVD	Literature ([59,63])	HLA-DRB1*04:01, HLA-DRB1*15:01
LKFYSKISEYRHYCY	Literature ([64])	HLA-DRB1*01:01
MLDLQPETTDLYCYE	Literature ([64])	HLA-DRB1*01:01
TLRLCVQSTHVDIRT	Literature ([64])	HLA-DRB1*01:01
VFEFAFKDLFVVY	Prediction	HLA-DQA1*01:01, HLA-DQB1*05:01, HLA-DPA1*01:03, HLA-DPB1*04:01, HLA-DPA1*02:01, HLA-DPB1*05:01, HLA-DPA1*01:03, HLA-DPB1*02:01
ELTEVFEFAFKDLFVVY	Prediction	HLA-DPA1*01:03, HLA-DPB1*04:01, HLA-DQA1*01:01, HLA-DQB1*05:01, HLA-DPA1*02:01, HLA-DPB1*05:01
LTEVFEFAFKDLF	Prediction	HLA-DPA1*02:01, HLA-DPB1*05:01, HLA-DPA1*01:03, HLA-DPB1*02:01, HLA-DPA1*01:03, HLA-DPB1*04:01
TVLELTEVFEFA	Prediction	HLA-DQA1*05:01, HLA-DQB1*02:01, HLA-DPA1*01:03, HLA-DPB1*04:01
LRAFQQLFLNTLSFV	Prediction	HLA-DPA1*01:03, HLA-DPB1*04:01, HLA-DPA1*02:01, HLA-DPB1*01:01, HLA-DPA1*03:01, HLA-DPB1*04:02, HLA-DPA1*02:01, HLA-DPB1*05:01
DLRAFQQLFLNTLSFVC	Prediction	HLA-DPA1*01:03, HLA-DPB1*04:01, HLA-DPA1*02:01, HLA-DPB1*01:01, HLA-DPA1*03:01, HLA-DPB1*04:02, HLA-DPA1*01:03, HLA-DPB1*02:01
ESSADDLRAFQQLFLNTL	Prediction	HLA-DPA1*02:01, HLA-DPB1*01:01, HLA-DPA1*01:03, HLA-DPB1*04:01, HLA-DPA1*03:01, HLA-DPB1*04:02
EARIELVVESSADDL	Prediction	HLA-DQA1*03:01, HLA-DQB1*03:02, HLA-DQA1*05:01, HLA-DQB1*02:01

**Table 3:** Population coverage analysis for the combined HLA class I and class II epitope dataset.

Class I		
Coverage	Average Hit	PC90
98.18%	6.48	2.47
Class II		
Coverage	Average hit	PC90
99.81%	15.29	9.03

**Table 4:** Synthetic long peptide constructs and their physicochemical properties.

FASTA Sequence	VaxiJen Score	Instability Index	GRAVY	Molecular Weight (Da)
MLDLQPETTDLYCYELLSVGGFAFKDLFVVYRLRMKMLDLQPETTDLYCYELLSVGGEYRHYCYSL	1.4279	37.45909	0.040909	7833.9792
LKFYSKISEYRHYCYLLSVGGLQDIEITCVYLRMKLTEVFEFAFKDLFLLSVGGFAFKDLFVV	1.1891	39.81286	0.590476	7415.6859
DRAHYNIVTFCCKCDLLSVGGFAFKDLFVVLRMKVFEFAFKDLFVVYLLSVGGLQDIEITCVY	1.0951	36.4	0.850794	7221.5035
DSTLRLCVQSTHVDLLSVGGEYRHYCYSLLRMKLKFYSKISEYRHYCYLLSVGGFEFAFKDLF	1.0271	38.92873	−0.01429	7498.5943
TLRLCVQSTHVDIRTLLSVGGLQPETTDLYLRMKDRAHYNIVTFCCKCDLLSVGGFAFKDLFVVYR	0.9781	37.7	0.262121	7541.7734
MHGDTPTLHEYMLLSVGGFAFSDLYVVYLRMKELTEVFEFAFKDLFVVYLLSVGGLQPETTDLY	0.6547	35.02656	0.40625	7358.4145
MLDLQPETTDLYCYELLSVGGFAFKDLFVVYRLRMKDLFVVYRDSIPHAACHKCIDFYLLSVGGEYRHYCYSL	1.1275	37.96452	0.206849	8612.9278
LKFYSKISEYRHYCYLLSVGGFAFKDLFVVYRLRMKTVLELTEVFEFALLSVGGLQDIEITCVY	1.1195	39.20172	0.51875	7526.7864
DRAHYNIVTFCCKCDLLSVGGFAFKDLFVVYRLRMKLRAFQQLFLNTLSFVLLSVGGLQDIEITCVY	0.9761	39.02537	0.652239	7714.0804
TLRLCVQSTHVDIRTLLSVGGLQDIEITCVYLRMKDLRAFQQLFLNTLSFVCLLSVGGFEFAFKDLF	0.9172	39.97463	0.650746	7644.9508
MLDLQPETTDLYCYELLSVGGLQDIEITCVYLRMKESSADDLRAFQQLFLNTLLLSVGGFAFKDLFVVYR	0.9848	39.74571	0.331429	8031.2119
LKFYSKISEYRHYCYLLSVGGFAFKDLFVVYRLRMKEARIELVVESSADDLLLSVGGEYRHYCYSL	0.9481	37.32894	0.084848	7811.9636
DRAHYNIVTFCCKCDLLSVGGFAFKDLFVVLRMKCVYCKTVLELTEVPAVLLSVGGEYRHYCYSL	1.0237	37.51538	0.550769	7401.7143
MLDLQPETTDLYCYELLSVGGRAHYNIVTFLRMKLTEVFEFAFKDLFLLSVGGSVYGDTLEK	0.8487	30.49839	0.227419	7099.1161
MLDLQPETTDLYCYELLSVGGKFYSKISEYLRMKLKFYSKISEYRHYCYLLSVGGLFLNTLSFV	0.982	39.17688	0.096875	7579.7813
LKFYSKISEYRHYCYLLSVGGYGTTLEQQYLRMKLKFYSKISEYRHYCYLLSVGGLNTLSFVCPW	0.9343	39.72492	−0.12615	7810.0122
MLDLQPETTDLYCYELLSVGGELTEVFEFALRMKVFEFAFKDLFVVYLLSVGGCPEEKQRHL	1.0156	39.10645	0.232258	7209.293
MLDLQPETTDLYCYELLSVGGHTMLCMCCKLRMKLTEVFEFAFKDLFLLSVGGTTLEQQYNK	1.013	33.57097	0.170968	7161.3828
ELTEVFEFAFKDLFVVYLLSVGGRFHNIAGHYRLRMKELTEVFEFAFKDLFVVYLLSVGGKLPDLCTEL	0.811	33.27	0.486	8022.4
MLDLQPETTDLYCYELLSVGGTIHDIILECVLRMKELTEVFEFAFKDLFVVYLLSVGGLIRCLRCQK	0.8199	36.09552	0.559701	7746.1327
TLRLCVQSTHVDIRTLLSVGGTPTLHEYMLLRMKLFMDSLNFVCPWCLLSVGGYAVCDKCLKF	0.5793	37.00794	0.573016	7138.5331
TLRLCVQSTHVDIRTLLSVGGRFHNIAGHYRLRMKCVYCKTVLELTEVPAVLLSVGGFAFKDLFVV	0.8791	38.62	0.5777	7392.82
TLRLCVQSTHVDIRTLLSVGGFYSRIRELRLRMKVFEFAFKDLFVVYLLSVGGDSAPILTAF	0.5897	36.52113	0.579032	7067.2829
MLDLQPETTDLYCYELLSVGGFAFKDLFVVYRLRMKLFMDSLNFVCPWCLLSVGGYMLDLQPETT	1.0507	38.34615	0.435385	7551.8357
MLDLQPETTDLYCYELLSVGGFAFKDLFVVLRMKLTEVFEFAFKDLFLLSVGGRFHNIAGHY	1.0857	39.02097	0.453226	7167.2824

**Table 5:** Three-dimensional structure analysis parameters for the 25 SLP set. MFR—residues located in the most favorable regions (%); AR—residues located in the allowed regions (%).

Peptide Number	QMEAN4	QMEAN Z-Score	%MFR	%AR
1	0.742796	−0.82878	94.8	5.2
2	0.723041	−0.99288	93	7
3	0.716242	−1.0865	96.5	3.5
4	0.723265	−0.9898	96.5	3.5
5	0.70044	−1.45772	94.9	5.1
6	0.766649	−0.36818	92.7	7.3
7	0.709004	−1.35817	96.9	3.1
8	0.697578	−1.32017	94.8	5.2
9	0.711653	−1.23814	91.8	8.2
10	0.730673	−0.96492	91.8	8.2
11	0.665429	−2.03666	90.5	9.5
12	0.682124	−1.72969	95	5
13	0.70809	−1.33992	98.3	1.7
14	0.681138	−1.58902	90.7	9.3
15	0.803581	0.140843	98.2	1.8
16	0.776045	−0.32012	93	7
17	0.745241	−0.69935	98.1	1.9
18	0.732722	−0.8731	92.7	7.3
19	0.813586	0.300254	91.8	8.2
20	0.737284	−0.86994	96.4	3.6
21	0.745382	−0.75361	95	5
22	0.701926	−1.43243	94.8	5.2
23	0.751108	−0.61793	94.5	5.5
24	0.747556	−0.74766	94.6	5.4
25	0.69386	−1.41245	92.6	7.4

**Table 6:** Molecular docking parameters of the 25 SLP set with TLR2.

Peptide #	HADDOCK Score	RMSD (Å)	Van Der Waals Energy (kcal/mol)	Electrostatic Energy (kcal/mol)	Desolvation Energy (kcal/mol)	Restraints Violation Energy (kcal/mol)	Buried Surface Area (Å^2^)
1	−129.3 +/− 1.3	0.5 +/− 0.3	−75.4 +/− 3.2	−261.7 +/− 16.1	−1.7 +/− 1.2	1.4 +/− 0.6	2336.5 +/− 53.2
2	−174.2 +/− 4.3	0.5 +/− 0.3	−64.8 +/− 4.5	−443.8 +/− 46.6	−20.6 +/− 2.9	1.0 +/− 0.4	2411.5 +/− 58.8
3	−156.7 +/− 10.1	0.5 +/− 0.3	−94.6 +/− 6.2	−246.2 +/− 16.2	−12.9 +/− 3.3	0.8 +/− 0.3	2486.8 +/− 106.0
4	−137.9 +/− 3.9	0.6 +/− 0.3	−62.0 +/− 4.0	−271.4 +/− 7.3	−21.7 +/− 1.4	0.6 +/− 0.4	2089.0 +/− 34.1
5	−132.0 +/− 0.9	0.5 +/− 0.3	−61.9 +/− 0.8	−273.5 +/− 22.7	−15.5 +/− 4.0	0.5 +/− 0.2	1816.7 +/− 39.4
6	−133.5 +/− 2.6	0.5 +/− 0.3	−63.4 +/− 4.6	−319.4 +/− 17.6	−6.3 +/− 3.3	0.8 +/− 0.2	2276.4 +/− 75.3
7	−178.7 +/− 1.0	0.5 +/− 0.3	−93.3 +/− 4.1	−395.1 +/− 21.0	−6.4 +/− 4.8	0.6 +/− 0.2	2908.0 +/− 62.4
8	−155.2 +/− 1.7	0.5 +/− 0.3	−58.9 +/− 2.2	−418.1 +/− 13.1	−12.7 +/− 2.6	0.6 +/− 0.2	1877.4 +/− 16.9
9	−134.6 +/− 1.6	0.5 +/− 0.3	−60.2 +/− 0.7	−281.3 +/− 3.6	−18.5 +/− 1.6	2.6 +/− 0.8	1962.7 +/− 49.6
10	−119.0 +/− 1.1	0.5 +/− 0.3	−59.3 +/− 6.2	−363.4 +/− 39.0	13.0 +/− 2.2	0.3 +/− 0.2	2003.0 +/− 27.2
11	−172.8 +/− 4.6	0.5 +/− 0.3	−86.0 +/− 4.6	−380.4 +/− 9.3	−10.8 +/− 1.9	0.4 +/− 0.1	2854.3 +/− 87.2
12	−166.8 +/− 2.8	0.5 +/− 0.3	−59.6 +/− 7.6	−589.7 +/− 40.7	10.6 +/− 3.0	2.1 +/− 0.7	2336.5 +/− 45.5
13	−167.5 +/− 2.7	0.5 +/− 0.3	−83.9 +/− 1.9	−275.0 +/− 23.9	−28.7 +/− 2.8	0.7 +/− 0.4	2405.8 +/− 69.8
14	−192.9 +/− 3.8	0.5 +/− 0.3	−105.8 +/− 6.0	−341.3 +/− 41.0	−19.0 +/− 1.3	1.0 +/− 0.5	2882.3 +/− 28.0
15	−164.9 +/− 7.0	0.5 +/− 0.3	−74.3 +/− 1.1	−339.0 +/− 37.4	−22.9 +/− 1.4	0.4 +/− 0.2	2470.6 +/− 64.7
16	−141.9 +/− 3.7	0.5 +/− 0.3	−90.1 +/− 4.8	−218.1 +/− 12.4	−8.3 +/− 3.7	0.5 +/− 0.3	2412.6 +/− 57.5
17	−139.8 +/− 1.5	0.5 +/− 0.3	−56.6 +/− 0.9	−309.2 +/− 17.9	−21.4 +/− 1.5	0.5 +/− 0.1	1756.1 +/− 24.2
18	−141.8 +/− 1.6	0.5 +/− 0.3	−67.6 +/− 3.0	−276.5 +/− 17.4	−19.0 +/− 2.5	0.7 +/− 0.8	1991.6 +/− 60.4
19	−164.9 +/− 2.0	0.6 +/− 0.3	−67.8 +/− 5.2	−430.8 +/− 17.3	−11.0 +/− 1.9	0.4 +/− 0.1	2209.3 +/− 87.2
20	−127.0 +/− 1.4	0.5 +/− 0.3	−61.9 +/− 2.3	−318.7 +/− 16.1	−1.4 +/− 3.2	0.7 +/− 0.4	2076.1 +/− 3.5
21	−160.3 +/− 1.2	0.5 +/− 0.3	−81.5 +/− 2.5	−294.2 +/− 6.3	−20.1 +/− 1.9	1.1 +/− 0.5	2217.8 +/− 29.3
22	−151.8 +/− 3.0	0.6 +/− 0.3	−76.2 +/− 6.8	−392.2 +/− 66.7	2.7 +/− 4.1	1.3 +/− 0.7	2470.0 +/− 84.7
23	−132.8 +/− 4.3	0.5 +/− 0.3	−65.7 +/− 2.0	−252.5 +/− 25.7	−16.7 +/− 2.2	0.7 +/− 0.2	1829.6 +/− 37.7
24	−155.2 +/− 1.4	0.5 +/− 0.3	−75.2 +/− 4.7	−336.1 +/− 28.6	−12.8 +/− 1.3	0.8 +/− 0.3	2092.6 +/− 68.3
25	−143.5 +/− 0.7	0.6 +/− 0.3	−75.5 +/− 2.5	−296.9 +/− 18.6	−8.7 +/− 3.0	0.8 +/− 0.1	2444.0 +/− 61.0

**Table 7:** Gibbs free energies (ΔG) and dissociation constants (K_d_) for TLR2-peptide complexes.

Peptide Number	ΔG (kcal/mol)	K_d_ (M)
1	−12.4	1.8 × 10^−9^
2	−14.1	1.2 × 10^−10^
3	−15.6	1.1 × 10^−11^
4	−12.5	1.6 × 10^−9^
5	−12.5	1.4 × 10^−9^
6	−12.1	2.9 × 10^−9^
7	−16.9	1.2 × 10^−12^
8	−10.3	5.3 × 10^−8^
9	−12.5	1.5 × 10^−9^
10	−12.1	3.0 × 10^−9^
11	−15.7	8.4 × 10^−12^
12	−12.7	1.1 × 10^−9^
13	−12.9	8.7 × 10^−10^
14	−14.5	6.2 × 10^−11^
15	−12.9	7.8 × 10^−10^
16	−14.1	1.1 × 10^−10^
17	−11.3	1.2 × 10^−8^
18	−13	6.4 × 10^−10^
19	−13.7	2.2 × 10^−10^
20	−12.7	1.0 × 10^−9^
21	−13.5	3.1 × 10^−10^
22	−13.7	2.2 × 10^−10^
23	−11.9	4.0 × 10^−9^
24	−13.8	1.9 × 10^−10^
25	−13	6.5 × 10^−10^

## Data Availability

All the data is provided in the article.
